# Real-life assessment of standardized contrast-enhanced ultrasound (CEUS) and CEUS algorithms (CEUS LI-RADS®/ESCULAP) in hepatic nodules in cirrhotic patients—a prospective multicenter study

**DOI:** 10.1007/s00330-021-07872-3

**Published:** 2021-04-15

**Authors:** D. Strobel, E.-M. Jung, M. Ziesch, M. Praktiknjo, A. Link, C. F. Dietrich, C. Klinger, M. Schultheiß, D. Jesper, B. Schellhaas

**Affiliations:** 1 Universitätsklinikum Erlangen, Friedrich-Alexander Universität (FAU) Erlangen-Nürnberg, Medizinische Klinik 1, Erlangen, Germany; 2 Universitätsklinikum Regensburg, Regensburg, Germany; 3 Diakonissenkrankenhaus Dresden, Dresden, Germany; 4 Universitätsklinikum Bonn, Bonn, Germany; 5 Universitätsklinikum Magdeburg, Magdeburg, Germany; 6 Caritas Krankenhaus Bad Mergentheim, Bad Mergentheim, Germany; 7 Klinikum Ludwigsburg, Ludwigsburg, Germany; 8 Universitätsklinikum Freiburg, Freiburg, Germany; 9 Universitätsklinikum Erlangen, Medizinische Klinik 1, Friedrich-Alexander Universität (FAU) Erlangen-Nürnberg, Ulmenweg 18, 91054 Erlangen, Germany

**Keywords:** Carcinoma, hepatocellular, Ultrasonography, Contrast media, Liver, Diagnostic imaging

## Abstract

**Objectives:**

Hepatocellular carcinoma (HCC) can be diagnosed non-invasively with contrast-enhanced ultrasound (CEUS) in cirrhosis if the characteristic pattern of arterial phase hyperenhancement followed by hypoenhancement is present. Recent studies suggest that diagnosis based on this “hyper-hypo” pattern needs further refinement. This study compares the diagnostic accuracies of standardized CEUS for HCC according to the current guideline definition and following the newly developed CEUS algorithms (CEUS LI-RADS®, ESCULAP) in a prospective multicenter real-life setting.

**Methods:**

Cirrhotic patients with liver lesions on B-mode ultrasound were recruited prospectively from 04/2018 to 04/2019, and clinical and imaging data were collected. The CEUS standard included an additional examination point after 4–6 min in case of no washout after 3 min. The diagnostic accuracies of CEUS following the guidelines (“hyper-hypo” pattern), based on the examiner’s subjective interpretation (“CEUS subjective”), and based on the CEUS algorithms ESCULAP and CEUS LI-RADS® were compared.

**Results:**

In total, 470 cirrhotic patients were recruited in 43 centers. The final diagnosis was HCC in 378 cases (80.4%) according to the reference standard (histology 77.4%, MRI 16.4%, CT 6.2%). The “hyper-hypo” pattern yielded 74.3% sensitivity and 63% specificity. “CEUS subjective” showed a higher diagnostic accuracy (sensitivity, 91.5%; specificity, 67.4%; positive predictive value, 92%; negative predictive value, 66%). Sensitivity was higher for ESCULAP (95%) and “CEUS subjective” (91.5%) versus CEUS LI-RADS® (65.2%; *p* < 0.001). Specificity was highest for CEUS LI-RADS® (78.6%; *p* < 0.001).

**Conclusions:**

CEUS has an excellent diagnostic accuracy for the non-invasive diagnosis of HCC in cirrhosis. CEUS algorithms may be a helpful refinement of the “hyper-hypo” pattern defined by current HCC guidelines.

**Key Points:**

*• Contrast-enhanced ultrasound (CEUS) has a high diagnostic accuracy for the non-invasive diagnosis of hepatocellular carcinoma (HCC) in cirrhosis.*

*• The CEUS algorithm ESCULAP (Erlanger Synopsis for Contrast-enhanced Ultrasound for Liver lesion Assessment in Patients at risk) showed the highest sensitivity, whereas the CEUS LI-RADS® (Contrast-Enhanced UltraSound Liver Imaging Reporting and Data System) algorithm yielded the highest specificity.*

*• A standardized CEUS examination procedure with an additional examination point in the late phase, after 4–6 min in lesions with no washout after 3 min, is vital.*

**Supplementary Information:**

The online version contains supplementary material available at 10.1007/s00330-021-07872-3.

## Introduction

Hepatocellular carcinoma (HCC) can be diagnosed non-invasively in high-risk patients if the typical hallmarks of arterial hyperenhancement followed by a hypoenhancement in the portal venous phase or late phase are present upon contrast-enhanced imaging. Depending on the size of focal liver lesions (FLLs), both contrast-enhanced CT and MRI show high diagnostic accuracies, with a sensitivity in the range of 48–62% for lesions < 20 mm and 92–95% for those ≥ 20 mm (with a trend towards higher sensitivity of MRI compared to CT) and specificity ranging between 85 and 100% [1]. Some guidelines, such as the Asian Pacific Association for the Study of the Liver (APASL) guidelines, consider contrast-enhanced ultrasound (CEUS) to be equivalent to MRI or CT for the non-invasive diagnosis of HCC, whereas other guidelines, such as the European guidelines by the European Association for the Study of the Liver (EASL), recommend CEUS in cases of inconclusive CT/MRI or contraindications for those imaging modalities. The American HCC guidelines by the American Association for the Study of Liver Diseases (AASLD) do not recommend CEUS at all for the non-invasive diagnosis of HCC, but instead recommend multiphase CT or MRI. However, several studies and meta-analyses have demonstrated the high diagnostic value of CEUS for the characterization of FLLs. More recently, the wordings of some national guidelines (including the European guidelines by the EASL) have been refined and now require a contrast washout in CEUS of “late onset (> 60 seconds) and mild intensity” [1–5]. The LI-RADS® system (Liver Imaging Reporting and Data System) developed by the American College of Radiology (ACR) in order to improve the reporting and documentation of FLLs in high-risk patients has been widely used for several years [6, 7]. More recently, standardized algorithms have also been developed for CEUS, such as ESCULAP (Erlanger Synopsis for Contrast-enhanced Ultrasound for Liver lesion Assessment in Patients at risk) and CEUS LI-RADS® [7–12]. Although there is limited data on this topic so far, initial studies suggest high diagnostic accuracies for all of these algorithms, with positive predictive values (PPVs) of > 90% for LI-RADS® CT/MRI, CEUS LI-RADS®, and ESCULAP [7–16].

Recent studies suggest that washout in HCC often does not occur until the late phase of CEUS or may even be totally absent. The intensity of washout is typically mild in HCC, as opposed to other tumor entities like intrahepatic cholangiocarcinoma (iCCA) or metastases, which usually show strong washout with early onset [17–25]. However, there is still a debate on the standardized timing of the late phase in CEUS; essentially, the widespread habit of ending the CEUS examination in the late phase, after 2–3 min, can potentially lead to late washout in HCC being overlooked [26].

Although first studies suggest good diagnostic accuracy of the new standardized CEUS algorithms, these algorithms have not yet been validated in a prospective real-life setting [7–16].

## Purpose

The aim of the study was to assess the diagnostic accuracy of standardized CEUS for the non-invasive diagnosis of HCC in cirrhotic patients in a prospective, multicenter real-life approach. The study was designed to compare the diagnostic accuracy based on subjective interpretation by an experienced examiner, the definition according to current guidelines (“arterial phase hyperenhancement followed by hypoenhancement”), and the recently developed CEUS algorithms ESCULAP and CEUS LI-RADS® [7–12].

## Materials and methods

### Study design

**Fig. 1:**
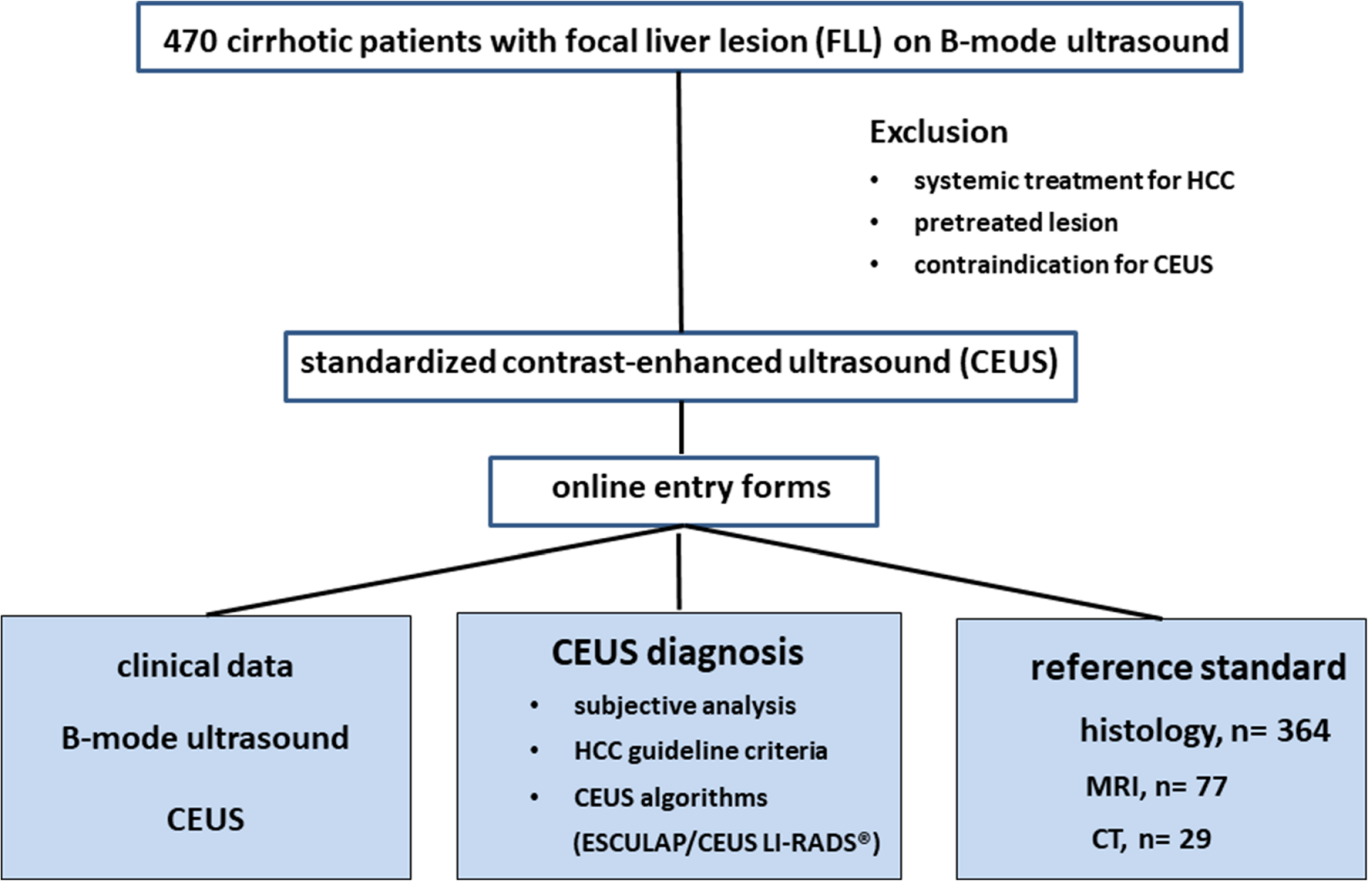
Study design with the inclusion and exclusion criteria, and the clinical and imaging data collected

From 04/2018 to 04/2019, patients at risk for HCC, as defined by national HCC guidelines, were recruited prospectively (cirrhosis of any origin, chronic hepatitis B infection, chronic hepatitis C infection with advanced fibrosis, non-alcoholic steatohepatitis/NASH, history of prior HCC). Inclusion criteria were age ≥ 18 years, the presence of a FLL visible on conventional B-mode ultrasound, and the availability of a reference standard. Histology was regarded as the gold standard; in cases with no available histological findings, MRI or CT was used as reference. Exclusion criteria were age < 18 years, systemic or local treatment for HCC, and contraindications for CEUS (such as known allergy or hemodynamic instability). The local ethics committee approved the study (ethics vote 16_17B). All patients provided their written informed consent according to DSGVO 05/2018 (European General Data Protection Regulation) for prospective evaluation of anonymized data. The study was initiated as a prospective nation-wide multicenter trial, registered as NIH trial (NCT03405909) and funded by the DEGUM (German Society for Ultrasound in Medicine). As liver cirrhosis is regarded as the main risk factor for HCC in all national HCC guidelines, we now present the data on patients with liver cirrhosis, which represents the majority of patients in our study cohort (> 90%). The study design is depicted in Fig. 1.

### Recruitment of participating centers

All centers with high expertise in abdominal ultrasound and CEUS (at least 6000–10,000 ultrasound examinations) were invited to participate in the study via the central registry of the German Society for Ultrasound in Medicine (DEGUM). Participating centers were equipped with individual, password-protected, online accounts with personal access to the online data entry forms. Before the start of patient recruitment, participating centers were invited to two meetings on the non-invasive diagnosis of HCC cirrhotic patients with contrast-enhanced imaging based on current guidelines [1–6]. The two meetings included practical training sessions on the standardized CEUS examination protocol, CEUS criteria for the non-invasive diagnosis of HCC based on the guidelines, and the CEUS algorithms, and technical aspects of the online entry forms. Data from medical history, clinical background, B-mode ultrasound, CEUS, and the reference standard (histology, MRI, CT) were entered into the online entry forms (Table 1).

**Table 1:** Information retrieved from online entry forms of the DEGUM CEUS HCC study. Summarizes the information collected via the online entry forms. Examiners collected clinical and imaging data according to the categories displayed and entered them via personalized password-protected accounts

**General information:** Participating center Ultrasound device used Examiner Date of examination Automatically generated case number	
**Patient characteristics:** Age Gender Risk factor for HCC Presence of liver cirrhosis Known extrahepatic malignancy Diabetes mellitus General condition (ECOG)	
**B-mode ultrasound:** Imaging quality Conditions of liver parenchyma (cirrhosis, steatosis, uncharacteristic changes, normal parenchyma) Presence of portal vein thrombosis Presence of transjugular intrahepatic portosystemic stent shunt (TIPS) Number of focal liver lesions Size of target lesion Echotexture of target lesion Presence of hypoechoic rim Macroinvasion of liver vessels Findings within Milan criteria [27]	
**CEUS:** Imaging quality Application of second contrast bolus Enhancement behavior of the index lesion relative to the surrounding parenchyma: In the arterial phase at 1 min at 3 min After 4–6 min in the case of no washout at 3 min Presence of enhancing tumor thrombus On-site diagnosis according to CEUS	
**Reference standard:** Histology: Histological findings from index lesion (including grading in case of HCC) Histological findings from liver parenchyma MRI findings CT findings	

Categorization of the index lesion according to the standardized CEUS algorithms ESCULAP and CEUS LI-RADS®

### B-mode ultrasound and contrast-enhanced ultrasound

All patients underwent conventional liver ultrasound, followed by immediate CEUS. A complete ultrasound examination of the whole liver was performed, with an additional color mode for liver vessels. The number of liver lesions was recorded, and the largest or best accessible lesion was chosen as the target lesion. The target lesion was characterized according to the parameters given in Table 1. CEUS was performed according to the EFSUMB (European Federation of Societies for Ultrasound in Medicine and Biology) guidelines [17]. A standardized protocol was used with continuous assessment of the arterial phase until the maximum contrast enhancement was reached in the lesion, followed by intermittent scanning with short sweeps through the lesion at the following time points: 1 min; 3 min; 4–6 min in case of no contrast washout after 3 min [26]. In the case of insufficient contrast enhancement in the late phase, examiners were instructed to apply a second contrast bolus with subsequent assessment of the late phase only. Examiners had to describe the contrast enhancement pattern in the arterial phase. Moreover, they had to decide on the onset (early = < 60 s, late = after 1–3 min, very late = after ≥ 4–6 min) and intensity (mild versus marked) of the washout.

### CEUS algorithms

The standardized algorithms are designed to categorize FLLs in high-risk patients according to defined criteria (such as lesion size and contrast enhancement behavior), resulting in a category that describes the risk of a given lesion being a HCC. With ESCULAP, there are six categories: ESCULAP-1 = definitely benign; ESCULAP-2 = intermediate probability of HCC, uncertain findings; ESCULAP-3 = definite HCC; ESCULAP-C = iCCA; ESCULAP-V = HCC with tumor invasion of the hepatic veins or portal vein; and ESCULAP-X = non-categorizable [7–11]. With CEUS LI-RADS®, there are eight categories: CEUS-LR-1 = definitely benign; CEUS-LR-2 = probably benign; CEUS-LR-3 = intermediate probability of malignancy; CEUS-LR-4 = probably HCC; CEUS-LR-5 = definitely HCC; CEUS-LR-M = probably or definitely malignant, not necessarily HCC; CEUS-LR-TIV = tumor in vein; CEUS-LR-NC = non-categorizable [7]. The ESCULAP algorithm is shown in Supplemental Figure 1. The CEUS LI-RADS® algorithm is displayed on https://www.acr.org/Clinical-Resources/Reporting-and-Data-Systems/LI-RADS/CEUS-LI-RADS-v2017.

### Diagnosis of HCC

For the diagnosis of HCC in a real-life setting, the following options were directly compared:

1. “Subjective interpretation of CEUS”: the examiner was asked to decide for or against HCC according to his/her subjective impression, based on a combination of experience and his/her impression from B-mode ultrasound, color mode, and CEUS, without further restrictions.
2. “CEUS according to HCC guidelines”: the diagnosis of HCC was made if the pattern of arterial phase hyperenhancement followed by contrast washout was present. For this definition, washout could be of any intensity and with onset in the portal venous or late phase (following the definition in the German HCC guideline).
3. “CEUS algorithms”: the diagnosis of HCC was based on the criteria defined for ESCULAP-3 and CEUS LI-RADS® LR 5 [8–11]; Supplemental Figure 1].

### Reference

The reference standard for the assessment of diagnostic accuracy of CEUS was histology. If histology was not available, contrast-enhanced MRI or contrast-enhanced CT was accepted as reference.

### Statistical analysis

Data was exported from the online entry forms using Microsoft Excel. Quantitative variables are summarized by mean and standard deviation (SD). Frequencies are shown for categorical variables.

For assessment of the reference standard, we considered the subgroup of 395 patients with histological findings, i.e., with available gold standard, and evaluated the hierarchical combination of MRI and CT.

Diagnoses were defined positive for HCC and negative otherwise. Negative diagnoses included iCCA, metastases from extrahepatic malignancies, and benign lesions. Sensitivities, specificities, PPVs, and NPVs are shown with 95% confidence intervals. The normal approximation interval was used for 95% confidence intervals. Imaging modalities were compared separately within groups of patients with HCC and without HCC by McNemar’s test [28]. The *p* values for comparison of modalities with respect to predictive values were estimated by the R-package DTComPair using the function pv.gs(), which uses the approach by Leisenring et al [29]. All *p* values below 0.05 were considered statistically significant. Analyses were performed in R 3.5.2 [30].

## Results

### Participating centers and patient characteristics

Patients were recruited prospectively in 43 centers (16 academic centers) with a total of 50 experienced examiners referred to as the CEUS HCC study group. Patient characteristics of the 470 cirrhotic patients are shown in Table 2. Most patients had compensated liver cirrhosis.

**Table 2:** Patient characteristics (*n* = 470 cirrhotic patients)

**Male/female (*n*; %)**	389/81 (82.8%/17.2%)
**Age [years] (mean ± SD)**	67.1 ± 10.3
**History of extrahepatic malignancy**	69 (14.7%)
**Diabetes mellitus**	174 (37%)
**General condition (ECOG)**	
ECOG 0	288 (61.3%)
ECOG 1–2	171 (36.4%)
ECOG 3–4	11 (2.3%)

Table 2 summarizes the clinical data of the 470 cirrhotic patients enrolled into the study

*SD*, standard deviation; *HCC*, hepatocellular carcinoma; *ECOG*, Eastern Cooperative Oncology Group performance status

### Reference standard of focal liver lesions

The final diagnosis was 378 HCCs and 92 non-HCC lesions (benign, *n* = 49; malignant, *n* = 43) based on the reference (*n* = 470). Histological findings were available in 364 patients (77.4%). MRI and CT served as reference standard in 77 (16.4%) and 29 (6.2%) cases, respectively. In cases with both MRI and CT available (*n* = 23), MRI was used as the reference. There were no inconsistencies in the diagnosis between MRI and CT in these patients.

### B-mode ultrasound and color mode

Findings from B-mode ultrasound and color mode are shown in Table 3. Most patients (58.3%) had solitary lesions; the mean size of the target lesion was 52 mm (range, 5–200 mm), with 84/470 lesions (17.9%) ≤ 2 cm.

**Table 3:** Findings from B-mode ultrasound and color mode (*n* = 470)

Number of lesions	
Solitary lesion	274 (58.3%)
2–3 lesions	106 (22.6%)
> 3 lesions	49 (10.4%)
Diffuse tumor infiltration	41 (8.7%)
**Size of index lesion (*n*** **= 470) [mean ± SD]**	52 ± 111
≤ 10 mm	11 (2.3%)
11–20 mm	73 (15.5%)
21–50 mm	244 (51.9%)
≥ 50 mm	142 (34.9%)
**Echo texture of index lesion**	
Hypoechoic	280 (59.6%)
Isoechoic	100 (21.3%)
Hyperechoic	90 (19.1%)
**Presence of hypoechoic rim**	128 (27.2%)
**B-mode findings within Milan criteria** [26]	220 (46.8%)
**Macroinvasion of liver veins or portal vein**	61 (13%)
**Portal vein thrombosis**	36 (10.3%)
**TIPSS**	9 (2.6%)

Table 3 shows findings from B-mode ultrasound and color mode in the 470 cirrhotic patients. Both ultrasound characteristics of the focal liver lesions and of the liver parenchyma were recorded

*SD*, standard deviation; *TIPSS*, transjugular intrahepatic portosystemic stent shunt

### Contrast-enhanced ultrasound (CEUS)

In 106 cases (22.6%), a second contrast bolus was applied for improved late phase analysis. As to the 378 HCCs within the study collective, correct diagnosis according to the current guidelines (“hyper-hypo” pattern) was applied in 315/378 cases, with a sensitivity of 74.3% and a specificity of 63% (Table 4). A subjective CEUS-based diagnosis of HCC was made by the examiner in 376 of the 378 HCCs in the study, with both a superior sensitivity (91.5%; *p* < 0.001) and specificity (67.4%; *p* < 0.001). Additionally, the PPV and NPV were superior for the subjective CEUS-based diagnosis compared to those for the guideline-based diagnosis (92% vs. 89.2%, *p* < 0.05; 66% vs. 37.4%, *p* < 0.001). Overall, 61 HCCs (13%) showed macroinvasion of the liver veins or portal vein: in 34 of these cases, CEUS revealed contrast enhancement of the tumor thrombus. In 46/378 HCCs (12.2%), there was a very late onset of washout beginning after > 4–6 min.

**Table 4:** Diagnostic accuracy of different modalities compared to the reference standard

Modality	*N* (HCC)	*N* (Non-HCC)	Sensitivity	Specificity	PPV*	NPV*
CEUS on-site (subjective)	378	92	91.5% [88.7%; 94.3%]	67.4% [57.8%; 77%]	92% [89.3%; 94.8%]	66% [56.4%; 75.5%]
CEUS guidelines (hyper-hypo)	378	92	74.3% [69.9%; 78.7%]	63% [53.2%; 72.9%]	89.2% [85.8%; 92.6%]	37.4% [29.8%; 45%]
ESCULAP	279	70	95% [92.4%; 97.5%]	51.4% [39.7%; 63.1%]	88.6% [85%; 92.2%]	72% [59.6%; 84.4%]
CEUS LI-RADS©	279	70	65.2% [59.6%; 70.8%]	78.6% [69%; 88.2%]	92.4% [88.7%; 96.1%]	36.2% [28.5%; 43.8%]

*Relating to a prevalence of 80%

Table 4 shows the diagnostic accuracies of the different modalities tested in direct comparison (CEUS subjective; CEUS following the guidelines (“hyper-hypo” pattern); and the two standardized CEUS algorithms, ESCULAP and CEUS LI-RADS®). The normal approximation interval was used for 95% confidence intervals

*HCC*, hepatocellular carcinoma; *PPV*, positive predictive value; *NPV*, negative predictive value; *CEUS*, contrast-enhanced ultrasound; *ESCULAP*, Erlanger Synopsis for Contrast-Enhanced Ultrasound for Liver lesion Assessment in Patients at risk; *CEUS LI-RADS©*, Contrast-Enhanced UltraSound Liver Imaging Reporting and Data System

**Fig. 2:**
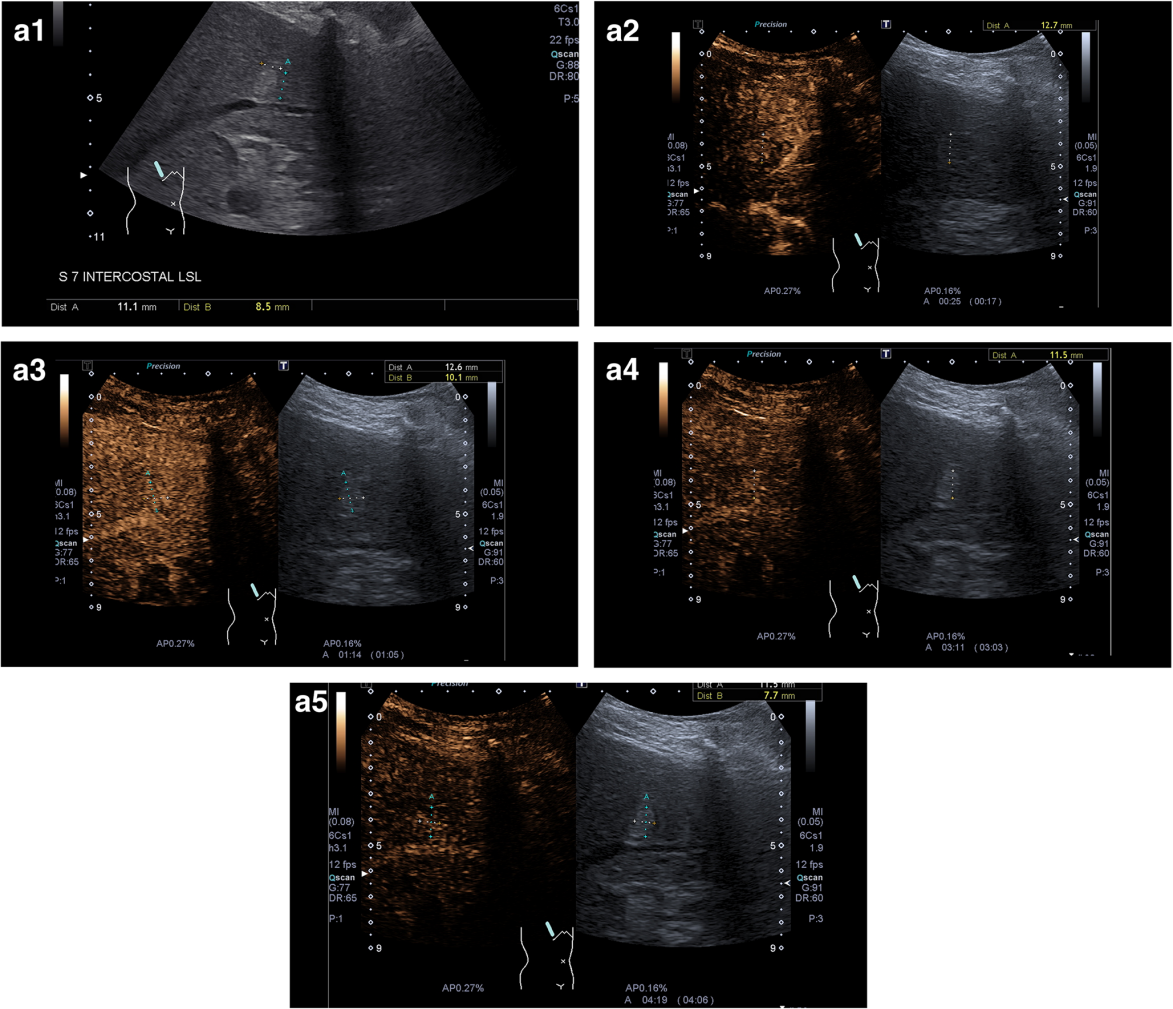

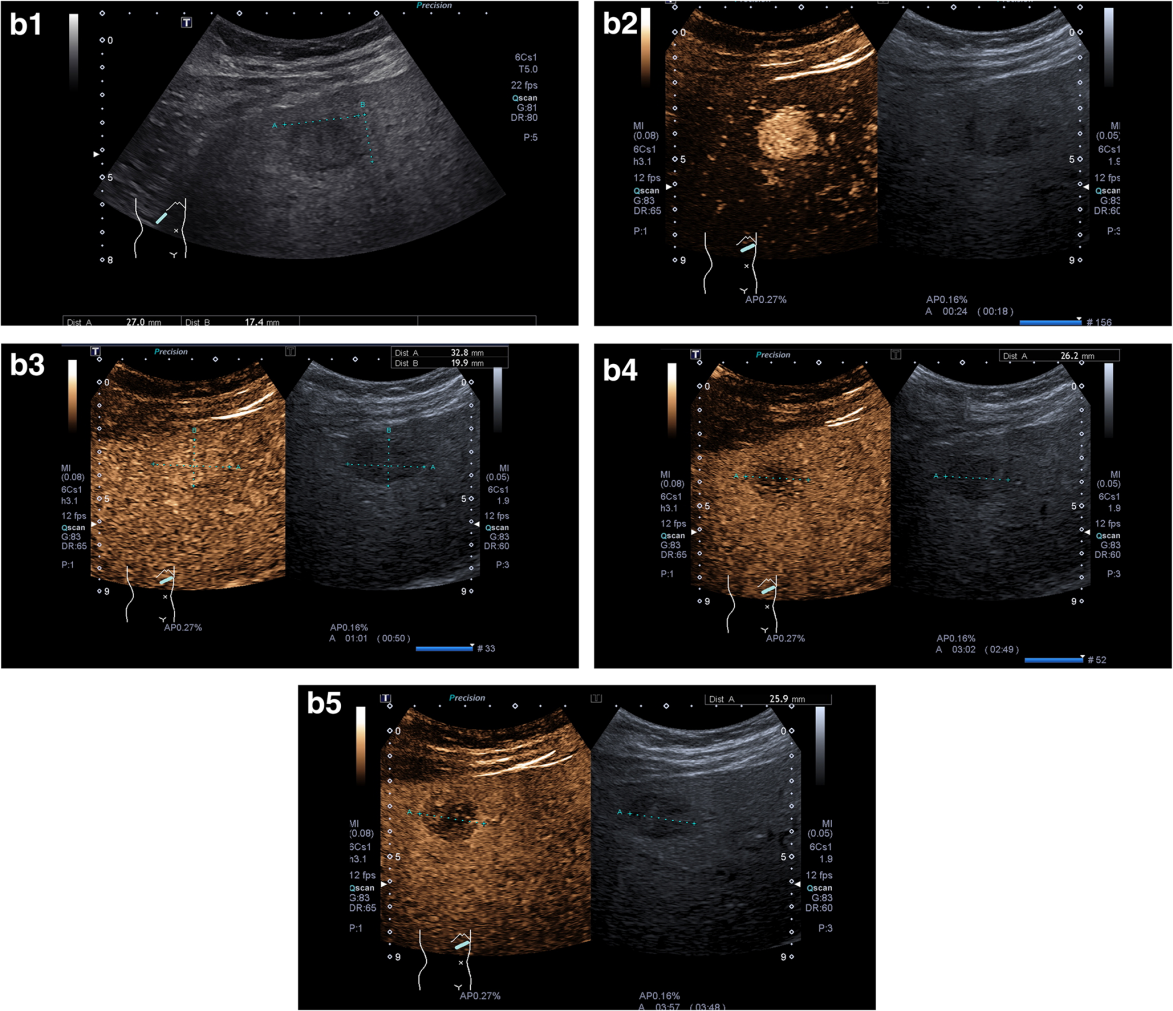

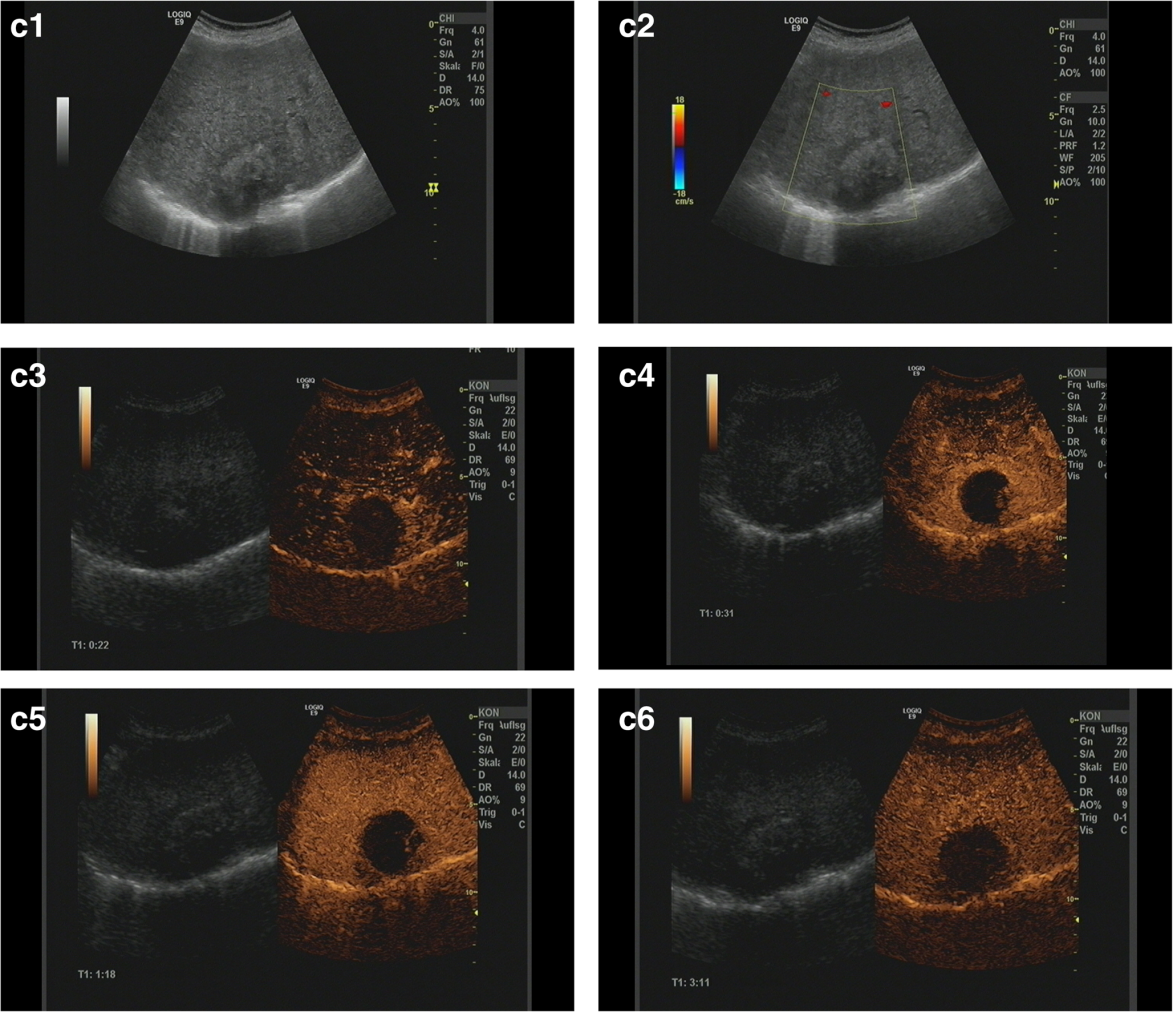
Representative CEUS images. **a** ESCULAP-1/CEUS-LR-3 lesion. **A1** B-mode: hyperechoic lesion of 11 mm in liver segment VII. **A2**–**A5** CEUS. The lesion shows arterial phase hyperenhancement (**A2**) with sustained hyperenhancement in the portal venous phase (**A3**) and late phase after 3 min (**A4**) and > 4 min (**A5**), corresponding to a benign lesion. **b** ESCULAP-3/CEUS-LR-5 lesion. **B1** B-mode: hypoechoic lesion of 27 mm in a cirrhotic patient. **B2**–**5** CEUS: homogenous hyperenhancement of the lesion in the arterial phase (**B2**), followed by isoenhancement in the portal venous phase (**B3**) and slight hypoenhancement in the late phase after 3 min (**B4**) and > 4 min (**B5**). **c** ESCULAP-C/CEUS-LR-M lesion, histologically HCC G2 in cirrhotic liver. This lesion was misclassified by both algorithms. **C1** B-mode: almost isoechoic lesion of 25 mm with a hypoechoic rim. **C2** Color mode: no hypervascularization visible. **C3**–**C6** CEUS: in the arterial phase, the lesion shows a rim enhancement with central hypoenhancement (**C3**–**C4**), followed by rapid and marked contrast washout in the portal venous phase (**C5**) and late phase (**C6**). Typical finding of iCCA; however, histologically proven HCC

The CEUS algorithms ESCULAP and CEUS LI-RADS® were applied in 349 cases of the 470 patients enrolled in the study (74.3%). In one case, the category of “non-categorizable” was chosen for both algorithms (ESCULAP-X/CEUS-LR-NC). The diagnosis according to ESCULAP was HCC (ESCULAP-3, ESCULAP-V) in 299 cases and iCCA (ESCULAP-C) in 14 cases. According to CEUS LI-RADS®, HCC (CEUS-LR-5, CEUS-LR-TIV) was diagnosed in 197 cases and other malignancies, including iCCA (CEUS-LR-M), were diagnosed in 40 cases, while 77 lesions were categorized as CEUS-LR-4 (probably HCC). In 349 cirrhotic patients with results for both algorithms, 181 of the 279 HCCs (64.9%) were correctly identified with both algorithms. In 84 HCCs (30.1%), correct diagnosis was made with ESCULAP only; in one HCC (0.4%), only CEUS LI-RADS® was correct. A further 13 HCCs (4.7%) could not be diagnosed by both algorithms. Typical CEUS images of the different categories are shown in Fig. 2.

### Diagnostic accuracy of different modalities

Data on diagnostic accuracy of the different imaging modalities is summarized in Table 4 for all 470 patients; in the case of the CEUS algorithms, only the subcollective of 349 patients with data available on both algorithms is shown.

Sensitivity for the diagnosis of HCC in high-risk patients was best when using the CEUS algorithm ESCULAP and the subjective CEUS-based diagnosis at the time of the examination. A significantly lower sensitivity was reached when examiners used the current definition of the guidelines and the CEUS LI-RADS® algorithm (*p* < 0.001). However, CEUS LI-RADS® showed the highest specificity of all modalities tested (79.6%; *p* < 0.01). The PPVs of all modalities, which refer to a prevalence of 80.4% in our patient collective, were in the range of 88–92% for all modalities, with a tendency towards slightly superior results for “CEUS subjective” and CEUS LI-RADS® compared to “CEUS subjective” and ESCULAP (*p* < 0.05). The highest NPV (72%) was found for the ESCULAP algorithm (*p* < 0.01).

## Discussion

According to the current European HCC guidelines, CEUS is recommended as a second-line imaging technique for the non-invasive diagnosis of HCC in patients at risk, when CT and MRI are contraindicated or are inconclusive for the HCC diagnosis. Our study provides prospective real-life data of CEUS in cirrhosis in a multicenter setting with a high number of patients. Our results reaffirm the excellent diagnostic accuracy of CEUS for the non-invasive diagnosis of HCC in cirrhotic patients in a prospective multicenter real-life setting. However, our findings suggest that the current definition of “hyper-hypo” pattern, as recommended by the national HCC guidelines, results in both lower sensitivity and specificity compared to subjective interpretation of CEUS by an experienced examiner. This superior performance can be explained by the fact that all examiners invited to participate in this study were highly experienced in abdominal ultrasound and CEUS, which partly explains the excellent diagnostic accuracy of “CEUS subjective.”

However, a high examiner expertise might not be available everywhere. Therefore, the CEUS algorithms might be helpful to standardized documentation and reporting. Our study showed that the sensitivity of “CEUS subjective” was surpassed only by the standardized CEUS algorithm ESCULAP, which at the same time showed the highest NPV of all modalities tested in this study. This is due to the fact that, contrary to the current guidelines and to the definition “definite HCC” with the CEUS LI-RADS® algorithm, HCCs without the characteristic “hyper-hypo” pattern can also be classified as “definite HCC” under certain circumstances with ESCULAP: for example, lesions ≥ 1 cm in size with arterial phase hyperenhancement only and no washout, or diffusely infiltrating tumors (Supplemental Figure 1). Also, the ESCULAP algorithm takes into account particularities of diffusely infiltrating HCCs that do not display the characteristic “hyper-hypo”, a fact which any experienced on-site examiner will also bear in mind and thus diagnose “HCC” in some cases, even in the absence of a “hyper-hypo” pattern. About 30% of definite HCCs were “undercategorized” when using CEUS LI-RADS®. These results are supported by a recent retrospective study in five Italian centers. Terzi et al found a sensitivity of 62% for CEUS LI-RADS® (LR-5) for the definite diagnosis of HCC [13]. In a very recent retrospective study assessing CEUS LI-RADS® in 175 lesions ≤ 20 mm in 172 patients, Huang JY et al. [15] found a sensitivity of 73.3% for LR-5, but a specificity of 97.1%. HCCs were seen in 48% of LR-4 lesions (11/23), 77/79 LR-5 lesions (98%), and 15/20 LR-M lesions (75%). However, histological findings were available in 124 cases (70.9%), with CT or MRI used as a reference standard in the remaining cases. Correspondingly, Ling et al conducted a small retrospective analysis of 56 histologically proven FLLs ≤ 2 cm in high-risk patients (44/56 HCCs = 78.6%) and found a sensitivity of 72.7% (32/44) for CEUS LI-RADS® LR-5, with a PPV of 86.5% (32/37) [14]. In another very recent retrospective study assessing CEUS LI-RADS® in 1826 patients with 2020 liver lesions, Zheng et al found a diagnostic accuracy of 81% for LR-5, with a sensitivity of 75%, a very high specificity of 96%, and a PPV of 98% [31]. For LR-M, the authors found a very low PPV of 36%, with 224 of 354 LR-M lesions (63%) revealed to be HCCs [31]. However, comparability is limited, as the patient collective in this retrospective study was restricted to patients with chronic hepatitis B infection, whereas our study included patients with liver cirrhosis only. Our study reaffirms the low sensitivity findings of CEUS LI-RADS® in a prospective multicenter real-life setting. On the other hand, the strength of CEUS LI-RADS® is its high specificity, intended to avoid false-positive diagnoses. Our data support the fact that CEUS LI-RADS® showed the highest specificity of all modalities tested. Another recent development with the aim of improving the standardization in the interpretation of CEUS examinations is the use of automated quantification algorithms for the detection and characterization of FLLs. Although data on this subject is limited, studies suggest that these algorithms might be a helpful addition to the conventional interpretation of CEUS examinations [32]. Similarly, standardized CEUS algorithms such as ESCULAP and CEUS LI-RADS® might be seen as an addition to, rather than a replacement of, conventional interpretation of CEUS.

A key point of CEUS LI-RADS®, which differs from the current national HCC guidelines, is the definition of “washout” as washout with late onset (> 60 s) and of mild intensity. This definition has already been entered into the recent European HCC guidelines [1]. Given the poor performance of the “hyper-hypo” pattern without further refinement (as represented by “CEUS guidelines” in our data), the “typical” pattern of HCC needs further specification.

One purpose of the CEUS algorithms was to overcome this problem by defining the “typical CEUS pattern” of HCC by means of several distinct criteria. For ESCULAP, this leads to the fact that “atypical HCCs” with a hyper-iso pattern, for example, can be categorized as HCCs, resulting in a high sensitivity.

Application of the CEUS algorithms was optional in our study, so that the frequency of their use could allow conclusions to be drawn about the clinical feasibility of the algorithms. The fact that the algorithms were applied in about ¾ of cases seems to suggest that examiners are open to their use and eager to adopt new criteria for more refined diagnosis of HCC with CEUS.

Our results suggest that the advantages of the CEUS algorithms seem limited compared to subjective diagnosis by an experienced examiner. Although this was not the subject of our study, it is possible that the CEUS algorithms might be more helpful for less experienced examiners. A very recent study hints at the possibility that it is unexperienced examiners particularly who can take advantage from the standardized CEUS algorithms [33].

However, our study has several limitations. The focus on liver lesions with available histological findings may have introduced a certain selection bias. However, choosing histology as the reference standard seemed the best way to enable an objective assessment of diagnostic accuracies of the CEUS modalities. In addition, the study collective was restricted to patients with liver cirrhosis, as this is the main risk factor for HCC. Therefore, it is not possible to transfer our prospective results to non-cirrhotic patients. Furthermore, only examiners with high expertise in abdominal ultrasound and CEUS were invited to participate, which might have introduced a bias to the real-life-setting, as CEUS examinations are performed by both experienced and inexperienced examiners in routine clinical settings. However, we chose this approach to ensure a high quality of both CEUS images and well-founded diagnoses. Additionally, the CEUS algorithms were not applied in all cases. However, this enabled us to estimate the clinical practicability of the algorithms, as examiners were free to choose whether or not to work with the CEUS algorithms. In general, CEUS is not recommended for the detection or staging of HCC; as a result, it will never replace MRI or CT, despite its high diagnostic accuracy for the characterization of FLLs. General limitations of the method, for example, in obese patients or those with massive ascites, are due to the physical properties of the technique.

The strengths of our study are the high number of patients, the prospective real-life setting, and the multicenter approach.

## Conclusion

This CEUS HCC study is the first multicenter, prospective comparison of standardized CEUS modalities, including the new CEUS-based diagnostic algorithms, in cirrhotic patients in a real-life setting. Our results reaffirm the excellent diagnostic accuracy of CEUS for the non-invasive diagnosis of HCC in high-risk patients; however, the simplistic definition of the “hyper-hypo” pattern to diagnose HCC needs to be refined. A standardized CEUS examination protocol with an additional examination point in the late phase after > 4–6 min in lesions showing no washout after 3 min is vital. Also, a second contrast bolus for better analysis of the late phase should be readily applied if needed. The main advantages of the CEUS algorithm ESCULAP are its excellent sensitivity and NPV, whereas the CEUS LI-RADS® algorithm provides high specificity at the cost of low sensitivity. However, subjective diagnosis by an experienced examiner achieves an almost equal diagnostic accuracy compared to the CEUS-based diagnostic algorithms.

## Supplementary Information

ESM 1 (DOCX 422 kb)

## Abbreviations

AASLD: American Association for the Study of Liver Diseases
ACR: American College of Radiology
APASL: Asian Pacific Association for the Study of the Liver
CEUS LI-RADS®: Contrast-Enhanced UltraSound Liver Imaging Reporting and Data System
CEUS: Contrast-enhanced ultrasound
CT: Computed tomography
DEGUM: Deutsche Gesellschaft für Ultraschall in der Medizin (German Society for Ultrasound in Medicine)
EASL: European Association for the Study of the Liver
EFSUMB: European Federation of Societies for Ultrasound in Medicine and Biology
ESCULAP: Erlanger Synopsis for Contrast-enhanced Ultrasound for Liver lesion Assessment in Patients at risk
FLL: Focal liver lesion
HCC: Hepatocellular carcinoma
iCCA: Intrahepatic cholangiocellular carcinoma
MRI: Magnetic resonance imaging
NASH: Non-alcoholic steatohepatitis
NPV: Negative predictive value
PPV: Positive predictive value
SD: Standard deviation

## Declarations

### Guarantor

The scientific guarantor of this publication is Prof. Dr. Deike Strobel, Department of Medicine 1, University Hospital Erlangen, FAU Erlangen-Nürnberg, Germany.

### Statistics and biometry

Dr. Christine Gall, Institute for Biometrics, Epidemiology and Statistics (IMBE), FAU Erlangen Nürnberg, kindly provided statistical advice for this manuscript.

### Study subjects or cohorts overlap

Some study subjects or cohorts have been previously reported in Schellhaas B et al, Ultraschall Med. 2020 Jul 27. doi: 10.1055/a-1220-8561. This study is a prospective multicenter study. However, the former study focused on the subcollective of high-risk patients (including risk factors other than cirrhosis) with histological findings available only, whereas the current manuscript deals with the subcollective of cirrhotic patients only and includes MRI or CT as the reference standard in patients with no histological findings available.

### Methodology

• prospective

• diagnostic or prognostic study

• multicenter study
