# Supplementary figures and images for: Real-life assessment of standardized contrast-enhanced ultrasound (CEUS) and CEUS algorithms (CEUS LI-RADS®/ESCULAP) in hepatic nodules in cirrhotic patients—a prospective multicenter study
Source: Eur Radiol. 2021 Apr 15;31(10):7614–25. doi: 10.1007/s00330-021-07872-3 (PMC8452566; doi:10.1007/s00330-021-07872-3)

Supplemental Figure 1:

Supplemental Figure 1 shows the ESCULAP algorithm [8-11].


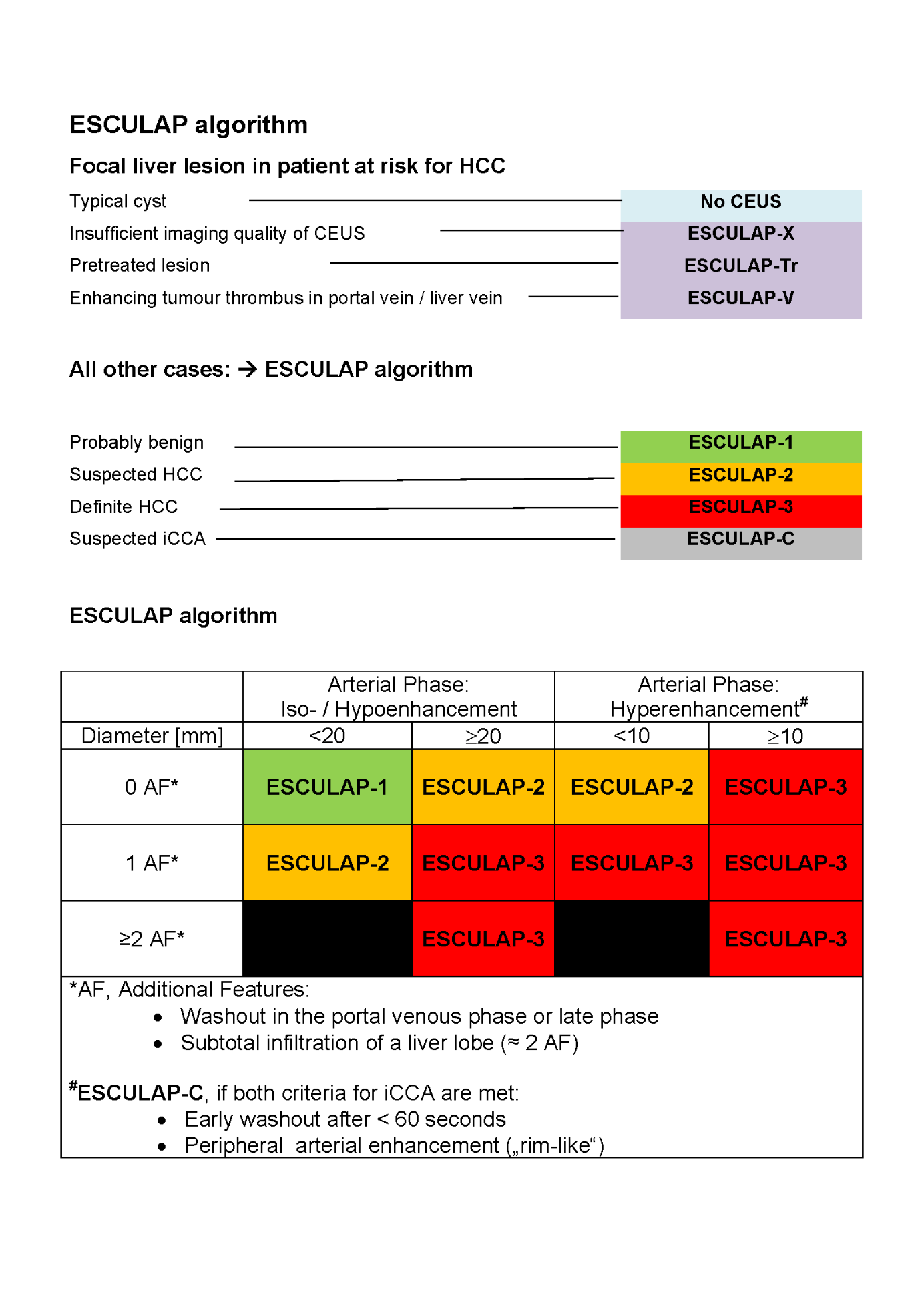

Supplement: Supplementary file 1 — (DOCX 422 kb) [file 330_2021_7872_MOESM1_ESM.docx]
